# NK Cell Development in Times of Innate Lymphoid Cell Diversity

**DOI:** 10.3389/fimmu.2020.00813

**Published:** 2020-07-08

**Authors:** Vladislava Stokic-Trtica, Andreas Diefenbach, Christoph S. N. Klose

**Affiliations:** ^1^Department of Microbiology, Infectious Diseases and Immunology, Charité–Universitätsmedizin Berlin, Berlin, Germany; ^2^Max-Planck Institute for Infection Biology, Berlin, Germany; ^3^Berlin Institute of Health (BIH), Berlin, Germany; ^4^Mucosal and Developmental Immunology, Deutsches Rheuma-Forschungszentrum, Berlin, Germany

**Keywords:** NK cells, innate lymphoid cells, immune recognition, immune receptor, innate lymphocytes

## Abstract

After being described in the 1970s as cytotoxic cells that do not require MHC-dependent pre-activation, natural killer (NK) cells remained the sole member of innate lymphocytes for decades until lymphoid tissue-inducer cells in the 1990s and helper-like innate lymphoid lineages from 2008 onward completed the picture of innate lymphoid cell (ILC) diversity. Since some of the ILC members, such as ILC1s and CCR6^−^ ILC3s, share specific markers previously used to identify NK cells, these findings provoked the question of how to delineate the development of NK cell and helper-like ILCs and how to properly identify and genetically interfere with NK cells. The description of eomesodermin (EOMES) as a lineage-specifying transcription factor of NK cells provided a candidate that may serve as a selective marker for the genetic targeting and identification of NK cells. Unlike helper-like ILCs, NK cell activation is, to a large degree, regulated by the engagement of activating and inhibitory surface receptors. NK cell research has revealed some elegant mechanisms of immunosurveillance, coined “missing-self” and “induced-self” recognition, thus complementing “non-self recognition”, which is predominantly utilized by adaptive lymphocytes and myeloid cells. Notably, the balance of activating and inhibitory signals perceived by surface receptors can be therapeutically harnessed for anti-tumor immunity mediated by NK cells. This review aims to summarize the similarities and the differences in development, function, localization, and phenotype of NK cells and helper-like ILCs, with the purpose to highlight the unique feature of NK cell development and regulation.

## Introduction

In the mid-70s of the last century, two groups independently reported the presence of small lymphocytes with non-MHC-restricted cytolytic activity against cells expressing tumor antigens in mice (1–4). Such “natural” killer (NK) cells, capable of cell-mediated, rapid cytotoxicity in a germline-encoded receptor-dependent fashion upon encountering of target cells, were observed in humans as well (5). NK cells remained the only subset of innate lymphocytes for two decades until an additional subset was discovered, which expressed the integrin α_4_β_7_, lymphotoxin (LT)α_1_β_2_, and lymphoid cytokine receptors. However, this newly described cell subset was giving rise to neither T-lymphocytes nor B-lymphocytes. They were named lymphoid tissue-inducer (LTi) cells because they were among the first cells to infiltrate lymph node anlagen during embryogenesis and hence are instrumental for the development of most secondary lymphoid tissues (6).

Furthermore, from 2008 onwards, several groups reported the discovery of new types of non-T and non-B lymphocytes which, like NK cells and LTi cells, require the transcriptional regulator *inhibitor of DNA-binding 2* (ID2) and the common gamma chain (γ_c_) of the cytokine interleukins (IL)-2, 4, 7, 9, 15, and 21 for their development and/or maintenance (7–21). These cells were termed “innate lymphoid cells” (ILCs), which constitute lineages of professional cytokine-producing cells that mirror T helper cells in the utilization of transcription factors (TFs) required to establish distinct patterns of lineage-specific cytokine production and effector functions. It became obvious that the different ILC populations resemble the functional diversity found in T helper cell subsets, thus establishing a complementary innate counterpart to T helper cells (22).

In connection with these findings of ILC diversity, a novel ILC nomenclature was proposed in 2013 and amended in 2018 (22, 23). In analogy to T cells, two principal subsets of ILCs can be distinguished: cytotoxic ILCs (i.e. conventional NK cells) and helper-like ILCs (i.e. ILC1, ILC2, and ILC3) (24, 25). The general division of NK cells and helper-like ILCs is supported by various findings. First, while there is a common progenitor to all innate lymphocytes, variably referred to as early innate lymphoid progenitor (EILP) (26) or innate lymphoid cell progenitor (ILCP) (27), a more restricted common helper-like innate lymphoid cell progenitor (CHILP) with reduced potential for helper-like ILC can only be found downstream of the bifurcation with the NK cell lineage. Second, all helper-like ILCs but not NK cells require GATA binding protein 3 (GATA-3) for their differentiation (28). Third, helper-like ILCs are remarkably tissue-resident cells, whereas NK cells are circulating cells (29–31). Finally, the use of inhibitory and activating receptors of the KIR and the Ly49 families was found in NK cells but not in ILCs. Thus, two principal lineages of innate lymphocytes exist: helper-like ILCs and cytotoxic ILCs.

In analogy to T cells, ILCs are divided into functional groups, based on TFs required for their development as well as their role in immune responses (22). NK cells are functionally important for immunity against tumors and intracellular pathogens *via* classical perforin-dependent, cell-mediated cytotoxicity and production of interferon-gamma (IFN-γ). ILC1s are an important source of IFN-γ and tumor necrosis factor (TNF) to trigger type 1 immune responses and limit intracellular infections. While NK cells and ILC1s are functionally both promoting type 1 immune responses, they are developmentally dependent on two evolutionary related T-box TFs: eomesodermin (EOMES) and T-box expressed in T cells (T-bet) (32). NK cells express both EOMES and T-bet, but their development is only strictly dependent on EOMES. NK cells develop in T-bet-deficient mice and have a relatively mild functional defect (16, 33, 34). In contrast, ILC1s express T-bet but not EOMES and do not develop in T-bet-deficient mice (21, 35, 36). ILC2s require GATA-3 and B-cell lymphoma/leukemia 11B (BCL11B) for development and produce type 2 cytokines, mostly IL-5, IL-9, and IL-13, as well as other effector molecules, such as amphiregulin, promoting worm expulsion and tissue remodeling (12–14, 17, 37–42). Group 3 ILCs include fetal LTi cells and can be further divided into two groups in adult mice based on CCR6 expression with different developmental requirements and effector mechanisms (43, 44). Both CCR6^+^ ILC3s and CCR6^−^ ILC3s are dependent on the TF RORγt and produce IL-22 to fortify the epithelial barrier against infections, damage, and genotoxic stress (45–51). CCR6^+^ ILC3s also produce IL-17 and protect from fungal infections, whereas CCR6^−^ ILC3s down-regulate RORγt and IL-22, up-regulate the TF T-bet. CCR6^−^ ILC3s in addition acquire the capacity to produce IFN-γ and transform into ILC1-like cells (19, 44, 52–55).

Helper-like ILCs were reported as tissue-resident cells enriched at barrier surfaces and underrepresented in secondary lymphoid organs (29–31). In contrast, NK cells are patrolling lymphocytes, which express CD62L to migrate from blood to lymph nodes (21, 30, 56). As patrolling cells, immune recognition by NK cells is mediated by the interaction of immunoreceptors that scan target cells for the expression of their ligands. Therefore, the development and regulation of NK cells depend on the interaction of the immunoreceptors and their ligands. Although the expression of some immunoreceptors (e.g. KLRG1, PD-1) has been reported for helper-like ILCs as well, their activity seems to be predominantly regulated by soluble factors such as cytokines and neuronal factors (21, 35, 52, 57–59).

## Immune Recognition Strategies of NK Cells

The complexity of multicellular organisms demands essential immune recognition strategies to maintain their self-integrity in a hostile environment. Almost all organisms, from bacteria to higher animals, possess recognition systems that allow them to discriminate between self and non-self and possess effector mechanisms to defend themselves from an invasion of pathogens. The immune system of vertebrates consists of two arms: innate and adaptive. Recognition of non-self molecules is broadly used by both the innate and the adaptive immune system to protect the host from infections (60). However, although NK cells are capable of directly sensing non-self molecules, their development and activation are regulated to a large extent by the recognition of self molecules. Discrimination between self and non-self is mediated by an array of stimulatory and inhibitory immunoreceptors expressed by NK cells. They either recognize non-self structures directly (Ly49H, NKG2C/CD94) or indirectly *via* binding immune complexes to Fc receptors. Alternatively, they interact with self MHC I (Ly49s and KIR, “missing-self” recognition) or with ligands absent on healthy cells (NKG2D and NKp30, “induced-self” recognition). The regulation of NK cells, which relies on cell surface immunoreceptor–ligand interactions, is complemented by cytokines, such as type I interferons, IL-12, IL-15, and IL-18 (61–64).

### Non-self Recognition

The recognition strategy of “microbial non-self” is approached differently from the innate and the adaptive immune system. The cellular components of the innate immune system express germline-encoded receptors, called pattern recognition receptors (PRRs), which come in two forms: transmembrane receptors and secreted receptors (60, 65). These molecules recognize conserved pathogen-associated molecular patterns (PAMPs) or microbe-associated molecular patterns (MAMPs). The secreted PRRs lead to the opsonization of microbes and label them for destruction either by the complement system or by phagocytosis. PRRs expressed on the cell surface of innate immune cells, such as Toll-like receptors (TLRs), lead to the activation of immune signaling pathways, which trigger inflammatory or antimicrobial effector responses (66, 67).

Upon recognition of PAMPs and MAMPs on microbes by antigen-presenting cells (APCs), phagocytosis and processing of antigens in the lysosomal compartment of these cells are triggered. T cells express strictly antigen-specific T cell receptors (TCRs) that are generated by somatic genetic recombination, thereby providing a vast repertoire of specificities. TCRs recognize self and non-self peptides presented on APCs via MHC molecules, providing “signal 1” for T cell activation. However, TCR ligation by itself is not sufficient for efficient T cell activation. It requires a co-stimulatory signal (“signal 2”), e.g. provided by APC-expressed CD80 (B7-1) and CD86 (B7-2), ligands for the constitutively expressed CD28 receptor on T cells (68). CD80 and CD86 are not expressed by unstimulated APCs but are rapidly up-regulated following the encounter of MAMPs or PAMPs, providing an additional “quality control” for T cell responses. In addition, stimulated APCs produce cytokines and IFNs, which further enhance T cell responses (“signal 3”). In the case of naive CD4^+^ T lymphocytes, distinct cytokines have been shown to drive the differentiation into one of three T helper (Th) subsets. IFN-γ and IL-12 are important for inducing Th1 cells, IL-4 for Th2 commitment, and TGF-β and IL-6 for Th17 cell differentiation (69–72).

**Figure 1:**
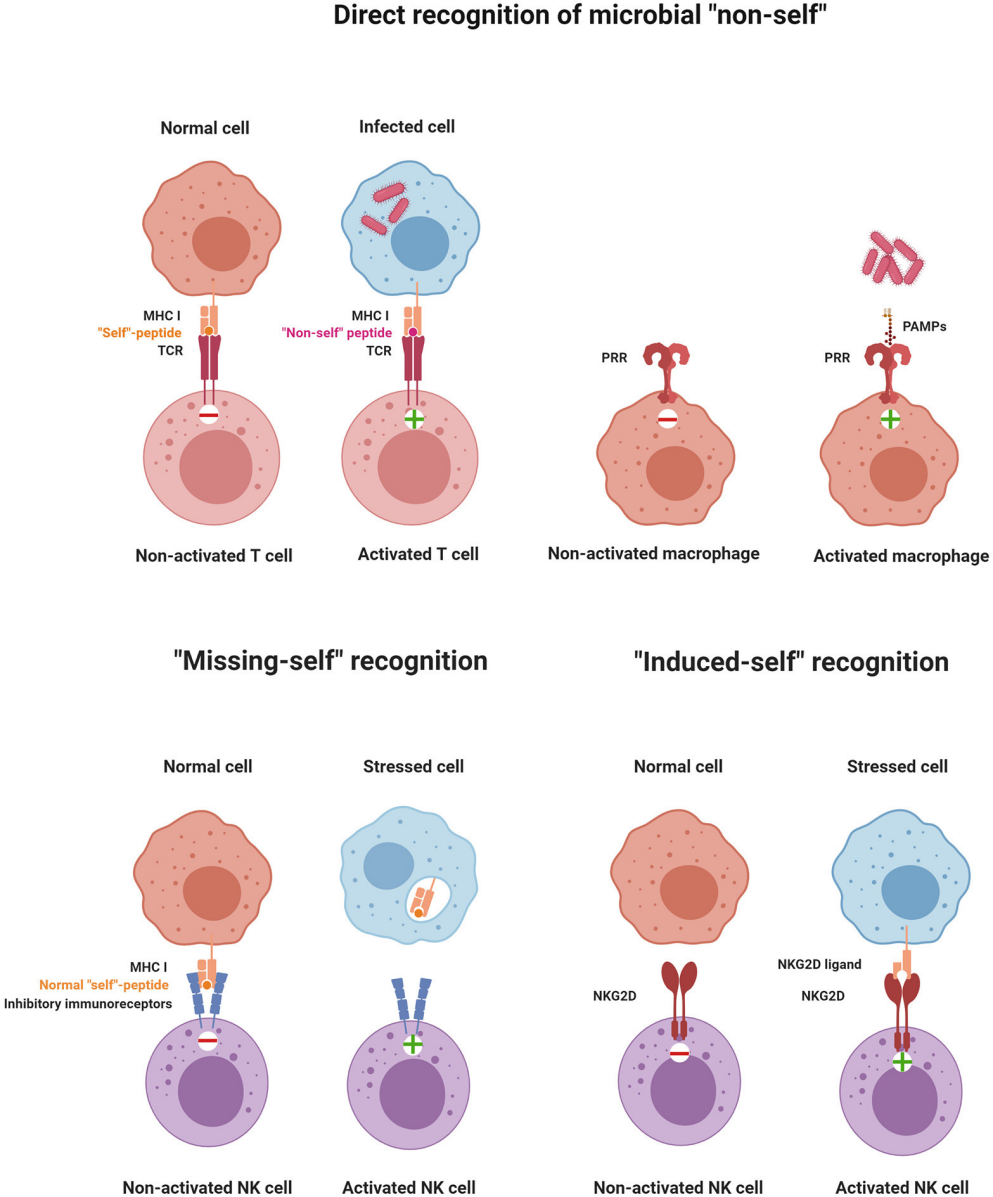
Principles of immune recognition. The immune system constantly senses the presence or absence of “self” and “non-self” molecules by stimulatory and inhibitory receptors. Activation of immune cells is triggered by the direct recognition of microbial “non-self,” “missing-self” recognition, or “induced-self” recognition (illustrations were created with BioRender.com).

TLR expression was also described on NK cells, but its contribution to NK cell activation remains unclear. While the direct activation of NK cells by TLR engagement has been reported for human NK cells (73), genetic data from mice demonstrated that TLR signaling to activate NK cells was cell-extrinsic *via* mononuclear phagocytes (62). While recognition of “non-self” *via* PRRs may not be central for NK cells, they express other families of receptors to directly recognize “non-self” molecules such as Ly49H in mice or NKG2C/CD94 in humans (74–76). Ly49H is a stimulatory receptor that recognizes the MHC-like protein m157 encoded by murine cytomegalovirus (MCMV), which is expressed in infected cells and confers host protection in C57BL/6 (B6) mice. It should be noted though that, in most inbred mouse strains other than B6, m157 binds to an inhibitory Ly49 receptor, leading to immune evasion. NK cells can also recognize non-self peptides in the context of non-classical MHC I molecules, very similar to the immune recognition strategy of T cells. For example, subsets of NK cells expressing the stimulatory receptor NKG2C/CD94 were shown to recognize the UL40 antigen of human cytomegalovirus presented in the context of the non-classical MHC-I molecule HLA-E, and this recognition activates NK cells (77). The different recognition strategies of immune cells are depicted in Figure 1.

### Missing-Self Recognition

In 1981, Klas Kärre formulated the missing-self hypothesis (78). Missing-self recognition was conceived as the capacity of NK cells to attack cells that fail to express sufficient levels of class I MHC molecules. This concept was discovered while investigating the role of class I MHC molecules in NK cell and T cell responses to tumor cells (79, 80). Given the role that the class I MHC antigen presentation pathway plays in the revelation of virally infected cells to CD8^+^ T cells, it is not surprising that many viruses have evolved mechanisms that interfere with this pathway, thereby binding CD8^+^ T cells to virus-infected cells (81). The missing-self hypothesis predicted that NK cells express inhibitory class I MHC-specific receptors and that the down-regulation of MHC-I expression on virus-infected cells or tumors would unleash NK cells from inhibition. Years after postulating “missing-self recognition,” various classes of inhibitory MHC class I-specific NK cell receptors were identified. Ly49 receptors in mice (82) and the structurally unrelated but functionally analogous KIR family of inhibitory receptors in humans (83, 84) directly interact with class I MHC molecules. Both human and mouse NK cells express the heterodimeric CD94/NKG2A receptor, which monitors class I MHC molecules by another mechanism. CD94/NKG2A recognizes a non-classical MHC class I molecule, HLA-E in humans and Qa-1b in mice, when loaded with peptides that are derived from the signal peptide of classical class I MHC proteins (85). The inhibitory receptors have an immune-receptor tyrosine-based inhibitory motif (ITIM) in their cytoplasmic domain. Upon ligand recognition, phosphorylation of the ITIM's tyrosine residue serves as a signal for recruitment of protein tyrosine phosphatases, SHP-1 and SHP-2, which inhibit cytotoxic activity by further dephosphorylating tyrosine residues that are critical for NK cell activation (86, 87).

Missing-self recognition does not require viral infection or a malignant transformation of target cells. Uninfected and untransformed cells can be lysed by NK cells, as demonstrated in NK cell-mediated rejection of F1 bone marrow grafts (88) and bone marrow of β2-microglobulin-deficient mice that do not express class I MHC on the cell surface (89). Since T cells are not capable of recognizing and killing cells that down-regulated class I MHC expression due to viral proteins that hijack their expression pathway, NK cells are able to compensate this immunological function *via* missing-self recognition of MHC-deficient target cells.

### Induced-Self Recognition

Induced-self ligands of NK cell receptors are molecules that are absent or only at a very low level expressed on normal cells but up-regulated on infected cells, stressed cells, or tumor cells as a marker of “abnormal self.” Induced-self ligands bind to stimulatory immunoreceptors on NK cells and mediate their activation, leading to the lysis of the target cell (63, 90). The activating NK cell receptor natural killer group 2D family (NKG2D) has served as a paradigm for understanding the recognition of induced-self antigens. NKG2D binds to several induced-self ligands. The mouse ligands include RAE1α, RAE1β, RAE1γ, RAE1δ, RAE1ε, H60 (H60a, H60b, and H60c), and MULT1 (91–96). These NKG2D ligands belong to a group of non-classical MHC I molecules and contain α1 and α2 extracellular domains with homology to class I MHC molecules. In humans, MHC class I polypeptide-related sequence A (MICA) and B (MICB) represent additional NKG2D ligands that are not present in mice (91). MICA/B possess an α3 domain homologous to class I MHC molecules, but they neither require β2-microglobulin for expression nor do they present peptides (90). Another example of “induced-self recognition” is the natural cytotoxicity receptor NKp30 which interacts with the B7-like self-ligand B7-H6, the expression of which is induced on transformed cells (97). Thus, immunosurveillance of induced-self ligands by immunoreceptors such as NKG2D and NKp30 allows the immune system to detect and eliminate cells that have undergone “stress.” These receptor–ligand pairs represent interesting targets of anti-tumor therapies. In Tables 1A and 1B, ligand–receptor interactions, as well as the functional consequences for NK cell activation are summarized.

**Table 1:**
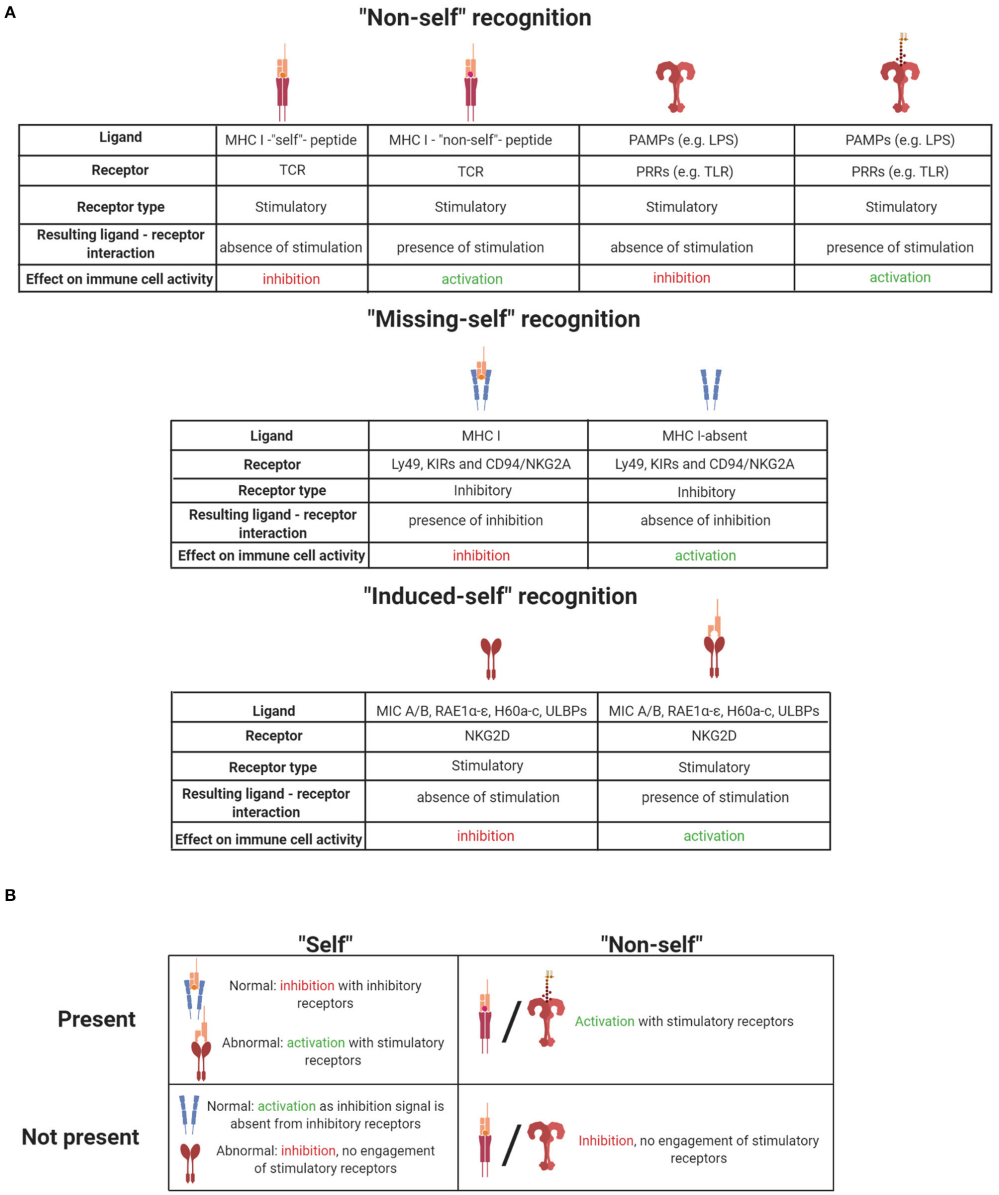
(A) Summary of ligand-receptor interactions and their effect on immune cell activation. (B) Summary of self and non-self effects on immune cells when they are present or absent. Illustrations created with BioRender.com.

Notably, NK cell-mediated immune regulation is tightly linked to both classical and non-classical class I MHC molecules. NK cells sense the absence of classical MHC-I (“missing-self”) but also recognize non-classical MHC-I molecules as non-self or induced-self ligands. In addition, NK cells require the recognition of self-MHC ligands not only for their activation but also for proper development, which will be discussed in the following chapters.

## Transcriptional Regulation of Progenitor Commitment to All Innate Lymphoid Cell Lineages

**Figure 2:**
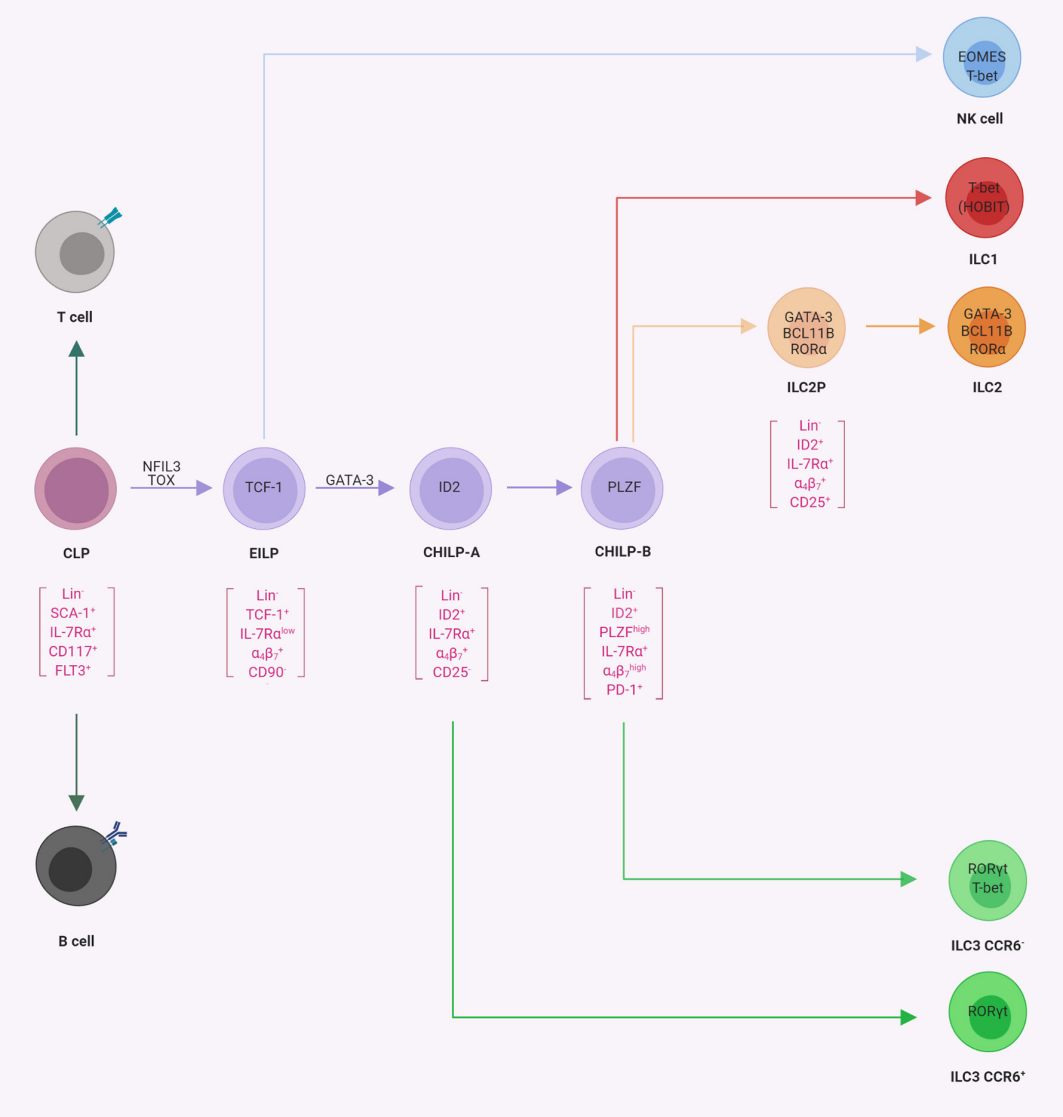
Progenitor commitment to innate lymphoid cells (ILCs). Schematic representation of progenitor populations with various differentiation potentials toward ILCs. The common lymphoid progenitor (CLP) gives rise to B-cells, T cells, and ILCs. The early innate lymphoid progenitors (26) possess the potential for NK cells, ILC1s, ILC2s, and ILC3s, whereas CHILP-A (21) and CHILP-B (98) possess the potential for ILC1s, ILC2s, and ILC3s as indicated. In square brackets are the population-defining markers reported in the literature. The transcription factors required for the indicated lineage or transition from one population to another are indicated within the cells or on the arrows, respectively (illustrations created with BioRender.com).

The initial steps of ILC differentiation from precursor cells take place in the fetal liver and after birth in the adult bone marrow (BM). In Figure 2, ILC progenitors and their differentiation stages into ILC subsets are presented.

Hematopoietic stem cells (HSCs) give rise to all blood cell progenitors, among which common lymphoid progenitors (CLPs) are precursors of all lymphocytes, belonging to both adaptive and the innate arms of the immune system. It is generally believed that the CLP differentiates into the various lymphocyte subsets by integrating environmental signals that establish characteristic transcriptional programs, usually regulated *via* several key TFs, that lead to a step-wise restriction of their precursor potential and to the instruction of lymphocyte subset-specific transcriptional circuitry (99–101).

An important conceptual advance of the last 5 years was the description of multipotent ILC progenitor cells such as α-lymphoid progenitor ($\alpha_{4}\beta_{7}^{+}\alpha$LP), EILP, CHILP, and ILCP, which have the developmental potential for ILC lineages but can no longer differentiate into adaptive lymphocytes or myeloid cells (21, 26, 27, 98). Early evidence indicated that ILC progenitors may be contained within a population with phenotypical characteristics similar to CLPs. Indeed Lin^−^ IL-7Rα^+^ CXCR6^−^ cells, which in contrast to CLPs expressed integrin α_4_β_7_ but were negative for FLT3, a receptor tyrosine kinase expressed by the CLPs (102), gave rise to all three groups of ILCs and T cells but had lost B cell potential (103). The subsequent acquisition of the chemokine receptor CXCR6 is indicative of the loss of T cell potential and Lin^−^ IL-7Rα^+^ CXCR6^+^ integrin $\alpha_{4}\beta_{7}^{+}$ FLT3^−^ cells are referred to as αLPs (103, 104). It became clear, however, that αLPs are a quite heterogeneous population of innate lymphocyte progenitors, which was further explored in subsequent work.

The earliest defined subset of ILC-committed progenitors downstream of the CLP (and contained within the αLP population) was characterized by high expression of the transcriptional regulator T cell factor 1 (TCF-1, encoded by the *Tcf7* gene). Such TCF-1^high^ progenitors are referred to as EILPs or ILCPs. EILPs already show a substantial expression of nuclear factor interleukin 3-regulated (NFIL3, also known as E4BP4) and thymocyte selection-associated high mobility group box protein (TOX) known to be involved in early ILC differentiation (see below) (26). While the CLP does not express ID2, a transcriptional regulator required for the differentiation of all ILCs (105), EILPs express intermediate level of ID2. The EILP gives rise to all ILC lineages (including NK cells) but lacks T and B lymphocyte or myeloid potential (26). Unlike other ILC progenitors and CLPs, EILPs were IL-7Rα low-expressing cells and developed independently of IL-7Rα signaling. Therefore this finding provoked the question on whether EILPs might constitute an alternative route to ILC development because the upstream and downstream cells are both IL-7Rα^+^. However, it was demonstrated in consecutive work that EILPs developed from CLP transiently down-regulating IL-7Rα expression and then differentiated into ILC progenitors with increased IL-7Rα expression (26, 106).

The existence of a common progenitor for ILCs was already hypothesized years before their discovery, mainly based on the phenotype of mice deficient for ID2 (107). ID2 belongs to the family of helix–loop–helix proteins, which form heterodimers with E-proteins, thus preventing their binding to DNA and antagonizing the gene regulatory function of E-proteins during cell development (108). Since *Id2*^−/−^ mice lacked all ILCs, it was hypothesized that this phenotype might be explained by the existence of a common ILC progenitor, which expresses ID2 and is developmentally dependent on it (12, 105, 109). Indeed the analysis of ID2 reporter mice [*Id2*^Gfp/+^ mice; (110)] revealed that both mature and immature ILCs expressed ID2 (17, 111, 112). An interrogation of the αLP population for ID2 expression revealed a population of ID2^high^ cells within Lin^−^ IL-7Rα^+^ CD25^−^ integrin $\alpha_{4}\beta_{7}^{+}$ FLT3^−^ αLP (21). Upon transfer and on a clonal level, this cellular subset gave rise to all three groups of ILCs, including CCR6^+^ ILC3, but not to conventional NK cells and was accordingly named CHILP. While the CHILP did not express any ILC lineage-defining TFs, they expressed intermediate levels of GATA-3 (17). However, CHILPs were heterogeneous for the expression of the TF promyelocytic leukemia zinc finger protein (PLZF) (21). Interestingly, while PLZF^+^ CHILP could generate ILC1, ILC2, and CCR6^−^ ILC3, they lacked the potential to differentiate into CCR6^+^ (LTi-like) ILC3s and NK cells (98). These findings might be explained by data showing that the CHILP contained subsets of CXCR5^+^ cells, which gave rise to CCR6^+^ ILC3s/LTi cells and were not contained in the PLZF^+^ population (113, 114). Single-cell sequencing of ILC progenitors has confirmed the developmental stages of early ILC commitment and further contributed additional markers, such as programmed cell death protein 1 (PD-1), to define PLZF^+^ precursors (115). Therefore, we will refer to these two populations of ILC precursors as CHILP-A (ID2^+^ PLZF^−^ PD-1^−^) and CHILP-B (ID2^+^ PLZF^+^ PD-1^+^). Using reporter mice for several TFs, later studies showed some degree of heterogeneity in CHILP-A and CHILP-B which could be further subdivided into cell subsets that were committed to one ILC subset and *bona fide* CHILP subsets that still maintained multi-ILC lineage potential (111–113).

A recent report attempted to challenge the view that ID2^high^ CHILPs are progenitors to all helper-like ILCs but not to conventional NK cells (112). This study was based on the generation of a very bright reporter allele for ID2. Somewhat expectedly (26), the authors found that ID2^int^ precursors (i.e. EILP), which could be discriminated in these new reporter mice, still have NK cell differentiation potential. These results are supporting previous work which show that EILP expressed intermediate levels of ID2 and can generate all ILC subsets and NK cells (26, 106, 116). It is worth noting that during cellular differentiation processes, TFs often act as gradients rather than as binary switches. Once past the NK cell bifurcation (Figure 2), ID2 expression increases (26, 100, 112) and ID2^high^ CHILPs have a more restricted potential. Collectively, the available data support a model of ILC differentiation downstream of CLP with three major bifurcations and consecutively restricted differentiation potential, namely, TCF1^+^ ID2^int^ EILP (or ILCP), ID2^high^ PLZF^−^ CHILP-A, and ID2^high^ PLZF^+^ CHILP-B (Figure 2).

Single-cell sequencing data and analysis of multi-color reporter mice have demonstrated that a further subdivision of ILC progenitors is technically possible. These findings can advance the ILC field by defining different ILC progenitors that have a more restricted differentiation potential and therefore open the perspective of finding the cues that control the commitment and the differentiation of CHILPs into different ILC cell subsets. However, defining progenitors by clustering based on highly expressed genes that are detected using single-cell sequencing comes with the caveat that cell clustering is not necessarily of biological relevance and differences in differentiation potential remain to be demonstrated.

Several additional TFs were recognized, which play an essential role in early commitment to the ILC fate. In addition to ID2 and PLZF, these TFs include NFIL3, TOX, and GATA-3. The phenotype of the knockout mice for these TFs was characterized by a deficiency or a reduction in all or almost all ILC lineages, except NK and LTi-like cells in GATA-3-deficient mice. NFIL3 is important for the transition from CLPs to ILC progenitors, where the relative expression of this TF increased and its deletion led to a substantial decrease in ILC progenitor numbers (104, 117–122). Since the down-regulation of the transcriptional regulator TOX was described in *Nfil3*^−/−^ mice in comparison to wild-type controls, it was proposed that NFIL3 is directly regulating the expression of TOX, which then acts downstream in ILC development (104). Indeed *Tox*^−/−^ mice had a similar phenotype as that of *Nfil3*^−/−^ mice and lacked mature ILCs and ILC progenitors (123, 124). Based on these data, it was proposed that NFIL3 and TOX orchestrate the transition from CLP to EILP (106), whereas GATA-3 (28, 125) is required later in ILC development for the transition to PLZF^+^ ILCPs. It should be noted though that NK cells and CCR6^+^ ILC3s still develop in GATA-3-deficient mice. Altogether these data indicate that the developmental potential for NK cell and CCR6^+^ ILC3s/LTi cell is consecutively lost during the transition from EILP to CHILP-B (28, 98, 114, 125, 126).

While ILC progenitors are certainly present in the primary organs of hematopoiesis, the BM and fetal liver (21, 26, 98), it should be considered that ILCs may be derived from local precursors as tissue-resident cells. In mice, fetal ILC precursors migrated to the intestine before Peyer's patch organogenesis and accumulated at the sites where intestinal lymphoid tissue organogenesis is initiated and became a localized source of ILC populations (127). While intestinal ILC precursors were identified based on arginase expression, adult BM ILC precursors lacked arginase expression, indicating tissue adaption of the ILC precursor population (127). Since fate-labeling studies suggest that the ILC pool is generated in different pre- and postnatal time windows (128) and ILC precursors were also detected in human blood (27), further research is required to investigate the relation among ILC precursor cells in different compartments and their relevance in ontogeny.

## Maturation OF Lineage-Committed NK Cell Precursors

### Developmental Stages of NK Cell Maturation

**Figure 3:**
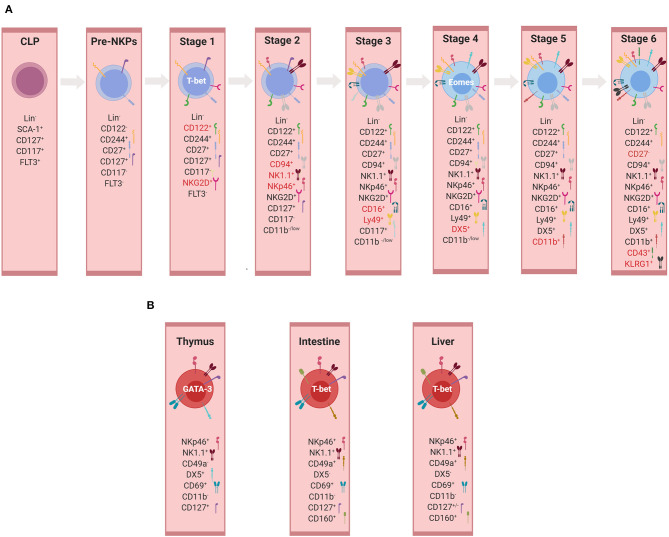
Development of NK cells and ILC1s. **(A)** Developmental stages of murine conventional NK cells. Representation of markers used for the identification of individual developmental stages. Markers highlighted in red represent those expressed mainly in the given subset and therefore important for its identification. At each stage, the expression of the listed markers is depicted as “+”, whereas the absence of expression is indicated by “–” or alternatively “low.” **(B)** Tissue-specific non-conventional NK/ILC1 subsets. Representation of markers used for the identification of non-conventional/ILC1 subsets in thymus, intestine, and liver (illustrations created with BioRender.com).

NK cells develop from a committed NK cell progenitor (NKP) in the BM, which was first described in 2001 based on the expression of the IL-2/IL-15 receptor beta chain (CD122), a well-recognized T-bet target gene (129, 130). In Figure 3A distinct developmental and maturation stages of NK cells are described. It should be noted that the major thrust of work on NK cell development was performed before ILCs with an NK cell phenotype (ILC1, subsets of ILC3) were recognized. We will critically discuss here the conventional definition of NKPs and immature NK cells in the new framework of ILC diversity.

An NKP population, which gave rise only to mature NK cells but did not possess a potential for T or B-cell lineages, was originally identified to be within Lin^−^ NK1.1^−^ DX5^−^ CD122^+^ cells (129). However, the frequency of such NKP differentiating into NK cells was only one in 12 in limiting dilution assays, revealing a highly heterogeneous population and a requirement for additional markers to further narrow down the true NKP, also because some T cell potential was still detectable in this population (131). Technical progress in multicolor flow cytometry allowed a more accurate definition of the NKP within Lin^−^ CD27^+^ CD244^+^ CD122^−^ IL-7Rα^+^ FLT3^−^ cells, in which 50% of the cells were giving rise to NKp46^+^ NK cells (132). This NKP subset was designated as a pre-NK cell precursor (pre-NKP), suggesting to be the earliest precursor of NK cells. Pre-NKPs express natural killer cell receptor 2B4 (CD244) and lack the expression of other surface markers associated with NK cells (NKp46, NKG2D, NK1.1, or inhibitory receptors such as Ly49 and CD94/NKG2A). Under NK cell-promoting culture conditions, the expression of CD122 is up-regulated, thereby giving rise to the ‘refined' NK cell precursor (rNKP), which has full NK cell potential (132). Apart from CD122, these precursors also acquire during their differentiation the IL-2 receptor γ_c_ chain, which makes NKPs responsive to IL-15, a cytokine essential for NK cell differentiation and survival (133–135). Both pre-NKPs and rNKPs showed the potential of differentiating into NK cell receptor-positive cells in spleen and liver, albeit with different frequencies. Since EOMES, a lineage-specifying TF for NK cells was not analyzed, these studies do not allow definite conclusions regarding a possible bipotential of NKPs for NK cells and ILC1s. The notion that the NKP population might contain committed ILC1 precursors was supported by Constantinides et al., who demonstrated heterogeneity within the pre-NKP population, with one subset expressing PLZF and other markers characteristic for ILC progenitors and another subset belonging to NKP (56). Therefore, more detailed analyses are required to delineate the separation of NK and ILC1 lineages in early precursors.

While NKG2D was already expressed by at least one subset of NKPs, the expression of NK1.1, NKp46, and CD94/NKG2A marked the immature NK cell stage (stage 2). The expression of Fc receptors and Ly49s, which provide inhibitory receptors for the NK cell education process, defines stage 3 of NK cell development (136–139). At this stage, “NK cells” display a T-bet signature, but since T-bet does not allow the distinguishing between NK cells and ILC1s, the delineation between the two lineages is difficult until EOMES and DX5 are expressed in stage 4 (16, 21, 35, 36). Furthermore, surface markers such as CD69, CD51, or tumor necrosis factor-related apoptosis-inducing ligand (TRAIL), which are often used to describe immature NK cells, are phenotypic markers of ILC1s but not mature NK cells in most tissues (136, 140, 141). Therefore, the view that the T-bet^+^ EOMES^−^ NKp46^+^ subset represents immature NK (iNK) cells is currently questionable (16, 142).

T-bet^+^ EOMES^−^ “NK cells” represent the major murine liver NK-like subpopulation during fetal and neonatal developmental stages. During aging, the ratio between these “T-bet^+^ EOMES^−^ immature NK cells” and mature NK cells changed, which represented another argument favoring the presumption that T-bet^+^ EOMES^−^ liver “NK cells” are in fact immature NK cells that can further mature. This hypothesis was supported by data demonstrating that EOMES^−^ TRAIL^+^ “immature” NK cells in the adult liver gave rise to EOMES^+^ DX5^+^ NK cells (16, 142). Contradicting results were obtained in several other publications (21, 35, 36). Daussy et al. utilized EOMES reporter allele and performed extensive phenotypic profiling of the EOMES-positive and EOMES-negative populations. Even though EOMES^−^ “NK cells” had an “immature phenotype” and EOMES^+^ NK cells had “mature” characteristics, *in vivo* and *in vitro* differentiation experiments did not show any transition between these two populations. PLZF fate mapping supported these results because EOMES^+^ DX5^+^ NK cells did not derive from PLZF-expressing progenitors, whereas EOMES^−^ TRAIL^+^ populations originated from PLZF^+^ precursors. In addition, EOMES^−^ TRAIL^+^ ILC1s did not differentiate into EOMES^+^ DX5^+^ NK cells (35, 36, 56). Moreover, Constantinides et al. demonstrated that ILC1s predominate over cNK cells during development in murine liver, while cNK cell number increases during adulthood (56). Therefore, since ILC1s and NK cells have parallel progression at an early stage during development and are phenotypically similar, further analyses have to be performed to separate iNK cells from ILC1s.

Mature *bona fide* NK cell subsets are characterized by the following: 1) expression of EOMES and DX5 (stage 4); 2) acquisition of CD11b (stage 5); 3) the consecutive loss of CD27 (stage 6) and the up-regulation of CD43 and KLRG1 (136, 143). Developmental intermediates were identified among CD11b^hi^ cells, designated as mature NK cell subsets (136). CD27 is a key marker of the NK cell lineage, dissecting the mature CD11b^+^ NK cell pool into two functionally distinct subsets (144, 145). The CD27^low^ NK cell subset possesses a higher threshold to stimulation and appears to be tightly regulated by the expression of NK cell inhibitory receptors. The preceding subset is consisting of the CD27^high^ NK cells that display a greater effector function, exhibiting a distinct tissue distribution and responsiveness to chemokines and productively interacting with dendritic cells (144, 145).

### Cytokine Signals Regulating NK Cell Development

Although NK cell commitment is not dependent on IL-2, IL-4, IL-7, IL-9, IL-15, or IL-21, which are executing their function through a common cytokine receptor γ chain, early NKPs have the capacity to respond to cytokines through the co-expression of CD122 and CD127 (146). Since mice lacking IL-2, IL-4, and IL-7 developed normal numbers of phenotypically mature NK cells with a regular capacity to exert natural cytotoxicity *in vitro*, produce IFN-γ, and kill tumor cells *in vivo* (146), IL-15 was identified as the major γ_c_ cytokine to promote NK cell development, and it plays a dominant role in early NK cell differentiation by maintaining normal numbers of immature and mature NK cells in the BM and spleen (146, 147). Given that the close association of T-box TFs and IL-15 responsiveness *via* CD122 is a hallmark of many lymphocytes, including ILCs and unconventional tissue-resident T cells (148–152), IL-15 was also indispensable for the development of ILC1s and ex-ILC3s although they co-express CD127 (21, 35, 153). IL-15 activated NK cells by STAT5 signaling and promoted the expression of the anti-apoptotic protein MCL1 and, at the same time, restricted the expression of pro-apoptotic proteins such as BIM and NOXA (153, 154).

### Transcriptional Regulation of NK Cell Development

NK cell development and function are regulated by a plethora of TFs expressed at different developmental stages, and at each stage these sets of TFs constitute regulatory networks for the establishment of distinct phenotypes. While numerous TFs that regulate pivotal steps during NK cell development were identified, the regulation of NK cell development is much less understood on a molecular level. As a consequence, the NK cell-specific target genes of TFs are insufficiently defined. Although TFs were proposed to mainly act during one stage of NK cell development, it should be considered that they might regulate NK cell development at various stages and depending on the amount of TFs being expressed (i.e. TF gradients). Additional difficulties in assembling the available data into a satisfying model are represented by the limitations in accurately recording relatively small NKP populations given the very limited availability of multicolor flow cytometry and by the separation of immature NK cells from ILC1s (as already discussed above). Therefore, although supported by data, the conclusion that certain TFs act at a certain stage of NK cell development should be taken with caution as these analyses often pre-dated the discovery of ILC1s and other ILC subsets expressing NK cell receptors such as NKp46 and NK1.1. With this in mind, we will discuss here the major TF modules that have been associated with NK cell differentiation.

Developmental defects in NK cells were reported from mice deficient for the TFs PU.1 and IKAROS that are broadly expressed early in hematopoiesis before commitment to the ILC/NK lineage and therefore affect multiple hematopoietic lineages, including but not limited to NK cells (155–158). Since it is controversial if PU.1 is expressed during NK cell development at all, it is unclear whether PU.1 or IKAROS are mediating effects during NK cell development or whether the phenotypes might be explained by the effects in upstream hematopoietic precursors (159).

Various TFs, such as TCF-1, NFIL3, and TOX, that were already introduced to regulate early commitment to the ILC lineage are indispensable for NK cell development, likely by acting on the EILP or upstream progenitors. Mice deficient for either TCF-1, NFIL3, or TOX lacked NK cells and also other ILC lineages (117, 123, 160). Since these TFs are already expressed upstream of the NKP in multipotent ILCPs such as EILP, CHILP-A, or CHILP-B, mice deficient in these TFs lacked most ILC lineages. Therefore, at least some of the effects likely occur already in multipotent precursors before commitment to the NK cell lineage (106, 124). Whereas, the mechanistic role for TOX after NK cell commitment is elusive, it was shown that TCF-1 restricts granzyme expression, thus protecting the developing NK cells from self-destruction (161). While NFIL3 was recognized as a TF important for the transition from CLPs to early ILC precursors, it was shown to be also up-regulated in the NKP cells. Further, its deficiency in mice led to decreased numbers of NK cells (121, 162). While it was proposed that NFIL3 regulates ID2 expression (117, 163), Seillet et al. showed that NFIL3 was influencing EOMES expression, whereas ID2 expression in the absence of NFIL3 remained the same. Moreover, ectopic expression of EOMES in *Nfil3*^−/−^ hematopoietic progenitor cells was sufficient to rescue cNK cell development (162).

Mice deficient for the TF ETS-1 had a strong reduction in mature NK cell numbers (164). Similar to NFIL3, ETS-1 was already expressed in CHILPs and also in NKPs. ETS-1 regulated the fitness of CHILPs, but the effects on NK cell development are probably emerging later with reduction at pre-NKP and rNKP stages, where ETS-1 might regulate T-bet and ID2 (165, 166). MEF is another member of the ETS TF family, which regulated essential functions during NK and NKT cell development, whereas B and T cells developed in normal proportions (167). While MEF-deficient mice have reduced NK cells and impaired effector function, including cytotoxicity and IFN-γ production, mechanistic insights are scarce (167).

Important regulators of NK cell development include ID2 and GATA-3, known to regulate early ILC commitment (21, 125). However, due to the phenotype of the gene-deficient mice and their expression pattern, it seems more likely that these TFs mediate their decisive effects after NK cell commitment. Mice deficient in ID2 or GATA-3 developed NKPs and immature NK cells but had a maturation defect of NK cells. In contrast, they lacked the other ILC lineages (12, 28, 105, 109, 126). While GATA-3 is required for ILC1 development (21, 28, 168, 169), it was dispensable for the development but not for the maturation of NK cells. GATA-3-deficient NK cells had an immature phenotype, were poor producers of IFN-γ, and showed defects in BM egress because they are retained in the BM due to the high CXCR4 expression (28, 126, 169). ID2 represents another transcriptional regulator that was up-regulated during NK cell development from NKPs and that was essential for the development of mature NK cells (105, 109, 162). Notably, unlike other ILC populations, NKPs and immature NK cells developed in ID2-deficient mice. However, ID2 deficiency causes loss of terminally differentiated CD11b^+^ NK cells, indicating a persistent need for the sequestration of E-proteins during NK cell maturation (170, 171). In support of this notion, the genetic deletion of ID2 and ID3, which both bind E-proteins, resulted in the complete loss of NK cells. It was also proposed that ID2 regulates IL-15 receptor signaling *via* the suppression of SOCS3. Interestingly, both ID2 and IL-15 signaling were linked to the regulation of apoptosis in NK cells *via* either anti-apoptotic MCL1 or pro-apoptotic BIM (154, 170, 172). Therefore, ID2 could be a link between sensing of the vital cytokine IL-15 and cell survival.

Several TFs with a more restricted expression during hematopoiesis played pivotal roles during the maturation of NK cells. These include EOMES, T-bet, and ZEB2. Unlike ILC1s, which only expressed and were developmentally dependent on T-bet but not EOMES, mature NK cells co-expressed both T-bet and EOMES. While mice with a conditional deletion of EOMES lacked NK cells, these cells normally differentiated in T-bet-deficient mice where they were accumulating in the BM and the lymph nodes due to the altered expression of S1P5R and CXCR3. They displayed an immature phenotype characterized by the persistent expression of CD27 and the reduced CD11b, CD43, and KLRG1 levels (33, 34, 173, 174). A similar NK cell maturation phenotype was reported from mice deficient in the Zinc finger-containing protein (ZEB2) (175). The notion that ZEB2 and T-bet might cooperatively regulate NK cell maturation is also supported by data showing that the overexpression of ZEB2 can partially rescue the phenotype of T-bet-deficient NK cells (175).

Although EOMES is also expressed by non-hematopoietic cells as well as in CD8^+^ T cells, where the TF regulates CD8 memory formation, among ILCs, EOMES represents a specific TF for NK cells (16, 176, 177). Moreover, mice harboring a conditional deletion of EOMES using NKp46^Cre^ completely lacked NK cells but still contain other ILC lineages (178, 179). Therefore, EOMES represents an attractive candidate for the specific targeting of NK cells by using, for example, *NKp46*^Cre^ *Eomes*^fl/fl^ mice to exclude effects on T cells. While epigenetic studies provide evidence that the EOMES and the T-bet promoters are both in an open chromatin configuration in NK cells, downstream targets of the T-box TFs are not well-defined in NK cells and were largely extrapolated from studies that have investigated other cell types (180). However, the importance of EOMES in NK cell fate and in the expression of prototypic markers of NK cells is also illustrated by data showing that the overexpression of EOMES under the *Tbx21* regulatory elements reprogrammed ILC1s to adopt phenotypical hallmarks of NK cells (178). Since the down-regulation of EOMES in NK cells mediated by TGF-β drove the NK cells to adopt an ILC1 phenotype, EOMES appears as a major signaling hub that dictate NK cell identity (181, 182).

Numerous TFs including AIOLOS, PRDM1 (BLIMP1), FOXO1, IRF2, RUNX3, and KLF2 regulate the late developmental stages of NK cells with main effects on terminal maturation and effector functions. PRDM1 (encoding BLIMP1) was shown to be regulated by T-bet and IL-15. Further, BLIMP1-deficient mice had fewer KLRG1^+^ mature NK cells. Although granzyme B expression was altered in PRDM1-deficient NK cells, effector functions, including cytotoxicity, remained normal (183). A similar phenotype was reported for mice deficient in the IKAROS zinc finger TF member AIOLOS. NK cells developed in AIOLOS-deficient mice but terminally differentiated CD11b^+^ NK cells were reduced. While NK cell effector functions were largely maintained, *Aiolos*^−/−^ NK cells were hyper-responsive to tumor cells, resulting in superior tumor surveillance (184).

The conditional deletion of the Krüppel-like TF KLF2 in hematopoietic cells using *Vav*^Cre^ resulted in the ablation of mature CD11b^+^ NK cells and consequently reduced the cytotoxicity toward target cells (185). It was proposed that KLF2 regulates the survival of NK cells *via* the regulation of IL-15 sensing and the expression of homing receptors. Reduced NK cells were also reported from mice deficient in the Th1 regulator interferon regulatory factor 2 (IRF-2) (186). IRF-2 deficiency disturbed mainly mature splenic NK cells, whereas NK cell development in the BM was only mildly affected. IRF-2 NK cells were more prone to undergo apoptosis during development independently from IL-15 (187).

TFs regulating NK cell development involves FOXO proteins as well. However, the precise role is hard to evaluate because of data that are difficult to reconcile with a model. While Wang et al. found decreased numbers of NK cells in mice with conditional deletion of FOXO1 using *NKp46*^Cre^ deleter mice (188), Deng and colleagues reported increased numbers of mature, hyper-reactive NK cells using *NKp46*^Cre^ and also *Vav*^Cre^ deleter mice to genetically ablate FOXO1 (189, 190).

Runt-related TFs (RUNX) are important regulators of lymphocyte development, including T cells and several ILC lineages. RUNX members 1–3 form heterodimers with the TF core-binding factor beta (CBF-β) in order to bind to regulatory DNA sequences and mediate gene transcription (191, 192). The RUNX3 isoform is highly expressed in NK cells. Different strategies were used to genetically interfere with RUNX to investigate the function *in vivo*, including the overexpression of dominant-negative RUNX3 or the conditional deletion of RUNX3 or CBF-β. While RUNX3 regulated the development of ILC1s and ILC3s by different mechanisms, ILC2 development remained intact and RUNX proteins protected ILC2s from an exhaustion-like phenotype (192–194). Concerning NK cell development, the deletion of either RUNX3 or CBF-β altered NK cell development *via* the regulation of CD122 and IL-15 responsiveness. This was accompanied by reduced numbers of CD11b^+^ and CD43^+^ mature NK cells and enhanced cytokine production (195–197). Consistent with a role later in development, RUNX and CBF-β were also crucial for NK cell memory formation following MCMV infection (198).

Finally, TFs that constitute regulatory network during NK cell development represent a nice example of how these proteins act as a part of a complex context that dictates their function and how compensatory mechanisms in their absence could, in some situations, buffer the entire system.

## Development Of ILC1s/Tissue-Resident NK Cells

NK cells and ILC1s share many phenotypical and functional properties that make the differentiation between these two innate lymphocyte subsets, especially in humans, very challenging (59). In addition, ILC1s comprise several subsets of lymphocytes previously referred to as “immature,” “tissue-resident,” or “unusual” NK cells before the revised nomenclature in 2013. These include TRAIL^+^ NK cells (142) and thymic NK cells (168) or (after the revised nomenclature) ILC1s in the BM, the lamina propria (21), the epithelium of the intestine [intraepithelial (ie)ILC1s] (20), the salivary glands (199), the adipose tissue (200), or the uterus (201). ILC1 subsets differ in terms of dependency on TFs during development, e.g. EOMES and NFIL3, and cytokines, e.g. IL-7 and IL-15 (21–23, 36, 120, 168, 202). Although these subsets are often all referred to as ILC1s, it is very difficult to conclude whether different developmental requirements reflect the tissue adaption of one cell lineage or different cell lineages of phenotypically similar cells. Besides the heterogeneity and the tissue adaptation of ILC1s, differences between mouse and human ILC1s add an additional layer of complexity to the topic. For example, it is well-established that murine liver TRAIL^+^ NK cells express and are developmentally dependent on T-bet but not EOMES. However, the human liver contains a population of CD56^bright^ lymphocytes, which phenotypically resembled ILC1s but expressed high levels of EOMES and only low levels of T-bet (203). Nevertheless, the functional and the phenotypical characterizations of different subsets of ILC1s are contributing to a better understanding of their biology and diversity as well as enabling their separation in a more comprehensive way. While being recognized as tissue-resident cells, ILC1s have been residing in various tissues, expressing specific markers that are represented in Figure 3B and which will be discussed below.

The characteristic feature of thymic non-conventional NK/ILC1 is that they express CD127 and developmentally depend on IL-7 signaling (168). This is in contrast to splenic and BM cNK cells, the phenotype and the function of which were not perturbed in the absence of IL-7. To a lesser extent, thymic NK cells required IL-15 for their development similar to NK cells (204). Moreover, thymic non-conventional NK cells depended on GATA-3 for their development and showed an elevated expression of this TF in comparison to splenic cNK cells (168). Phenotypically, thymic NK cells resembled ILC1s rather than NK cells because of the lack of CD11b and Ly49 receptors and their expression of CD69. However, whether thymic NK cells belong to the same lineage as ILC1s and TRAIL^+^ liver NK cells requires further clarification, especially because they express EOMES and DX5, which are usually not found on ILC1s (202). Thymic NK cells were reduced in *Foxn1*^−/−^ mice, which do not develop a functional thymus, suggesting that the thymus is an organ required for the generation of this ILC1 subset. Data obtained in reporter mice for TCR-δ germ-line transcription suggest that thymic NK cells might be derived from lymphocytes with T cell potential (205). This is in line with data showing that primitive, double-negative T cell progenitors still possess the potential to differentiate into cells that phenotypically resemble NK cells (206). Further, it was proposed that thymic NK cells might be the counterpart of CD56^bright^ NK cells, which are potent IFN-γ producers but have weak cytotoxic potential (168).

Based on CD56 expression, a unique subset of ILC1s was also described in the intestinal epithelium of humans. NKp44^+^ CD103^+^ and NKp44^−^ CD103^−^ ieILC1s were discriminated with similar functional properties, such as strong IFN-γ production. In addition, ieILC1s showed signs of TGF-β imprinting, such as CD103 expression, and were phenotypically different from cNK cells as illustrated by the expression of CD160, CD49a, CXCR6, CD69, and CD39, which were also found on ILC1s in other organs (141). Unlike thymic NK cells, ieILC1s lacked the expression of CD127 (IL-7Rα) but did express IL-2Rβ chain. The murine counterpart of human ieILC1s localizing within the gut epithelium co-expressed CD160, NKp46, and NK1.1 (20). Examining the developmental pathway of ieILC1s in mice, Fuchs et al. demonstrated the requirement of NFIL3 and T-bet. These ieILC1s were in part independent of IL-15Rα, indicating that intraepithelial ILC1s are developmentally distinct from cNK cells (20). Functionally, and similar to ex-ILC3s, ieILC1s were linked to the immunopathology in the αCD40 model of colitis due to their IFN-γ production (20, 52, 207). Further, ILC1s were enriched in patients with Crohn's disease and may, therefore, contribute to the development of inflammatory bowel disease similar to lamina propria ILC1s (19, 20, 208).

In the lamina propria of the intestine, it was challenging to identify ILC1s because of the sizeable populations of NK cells and ex-ILC3s, which all expressed the prototypic makers of ILC1s, such as NKp46 and NK1.1. Using double-reporter mice for EOMES (labeling NK cells) and fate-labeling for RORγt (labeling all ILC3s independent of their RORγt expression), a subset of lymphocytes within NKp46 and NK1.1 lymphocytes was defined, which expressed T-bet. This population within NKp46^+^ NK1.1^+^ lymphocytes lacked EOMES and RORγt expression and did not have a history of RORγt expression either. Further, such ILC1s were developmentally dependent on T-bet, NFIL3, and GATA-3, but not EOMES or RORγt. Phenotypically, intestinal ILC1s expressed markers associated with ILC1s in different tissues such as CD127, CD160, or CD49a, lacked markers of cNK cells such as CD11b and CD62L, and showed low Ly49 receptor expression. Despite expressing both CD127 and CD122, ILC1s were strictly IL-15-dependent and did not require IL-7. Upon transfer into alymphoid mice, ILC1s were a stable lineage without differentiation potential into cNK cells or ex-ILC3s and could also be found in the BM (35). BM ILC1s phenotypically overlap with the previously described immature NK cells based on markers such as CD69 (140). However, markers often connected to immature NK cells, such as CD69, TRAIL, or CD51, are rather found on ILC1s, and it should also be noted that they are not expressed before or after that developmental stage during NK cell development. In addition, CD69 is considered to be a marker for cell activation or tissue residency, which is associated with activated rather than with immature lymphocytes (136, 140). Therefore, additional studies have to address the potential heterogeneity within EOMES^−^ NK1.1^+^ cells, previously termed “immature NK cells” in the BM.

Although cytokine IL-12 was first described as a NK cell-stimulating factor (209), IL-12 elicited stronger effects on ILC1s than on NK cells, consistent with higher expression levels of the components of IL-12 receptor on ILC1s (21, 62, 210). While ILC1s were potent producers of IFN-γ and TNF, they expressed less perforin, indicating that they are less cytotoxic and rather mediate the cytotoxic effect by TNF receptors such as TRAIL. Functionally, a lack of perforin-mediated cytotoxicity or a loss of NK cell identity resulted in decreased immunosurveillance of tumors (181, 182, 211). However, data from different infection models suggest that there is a spatial and a temporal division of labor between NK cells and ILC1s. ILC1s protected the digestive tract from *Toxoplasma gondii, Clostridium difficile*, or MCMV infections, which are controlled to a large degree by IFN-γ secreted by ILC1s (21, 210, 212).

ILC1s, also referred to as tissue-resident NK (trNK) or TRAIL^+^ NK cells in the liver (142), differed from conventional NK cells since they expressed only T-bet as the key TF in mice, and this expression is favored in the liver microenvironment (16, 29, 35, 36). On the contrary, the BM provides a microenvironment that promotes lower expression levels of T-bet in NK cells, enabling the subsequent expression of EOMES (35). Another remarkable difference between cNK cells and ILC1s was the expression of the “homolog of BLIMP1 in T cells” (HOBIT) in ILC1s (213). This TF is specifically up-regulated in tissue-resident cells and controlled the expression of molecules associated with tissue residency, such as CD49a and CD69. Interestingly, HOBIT was essential for liver ILC1s but not for ILC1s in other organs investigated (210, 213). In addition, the development of ILC1s in the liver was demonstrated to be dependent on PLZF expression and independent of NFIL3, contrary to NK cells (36, 98).

TRAIL represents a prototypic marker of liver ILC1s as it is constitutively expressed on both mouse and human ILC1s, and together with CD49a and CD69, it has been used for separating liver ILC1s from NK cells. This type II transmembrane protein causes apoptosis primarily in tumor cells by binding to certain death receptors. Recent findings are suggesting that TRAIL expression is regulated by the activation of the NKp46 receptor in ILC1s since NKp46-deficient mice lack this effector protein (214–216).

Another important functional hallmark of liver pro-inflammatory ILC1s is that they are activated *via* IL-12, which are produced by conventional dendritic cells upon infection. After activation with IL-12, ILC1s respond with IFN-γ secretion to limit viral load and thereby contribute to early antiviral immunity at sites of primary viral infection (210). The genetic ablation of liver ILC1s is leading to increased MCMV load in mice; hence, NK cell responses are not the only early antiviral response in mice. In addition to rapidly responding to IL-12, “memory-like” qualities have been reported for ILC1s in models of contact hypersensitivity and MCMV infection. This is remarkable because these cells were originally considered as “immature NK” cells due to the lack of surface markers characteristic of mature NK cells (142). ILC1s were described to mediate tissue-resident memory responses to MCMV depending on glycoprotein m12 (217). Furthermore, previous reports have already linked liver ILC1s to memory responses during contact hypersensitivity reactions (29, 218). However, the mechanism underlying recognition of haptens by ILC1s following memory responses remains elusive.

Taken together, the experimental evidence obtained from knockout mice suggests that ILC1s constitute a separate tissue-resident lineage distinct from cNK cells. Further investigation is required to answer questions of ILC1 diversity.

## Epigenetic And microRNA-Mediated Regulation of NK Cell and ILC1 Development

Among epigenetic modifications, the deubiquitination of histone H2A by MYSM1 is important for NK cell generation as the deletion of this enzyme is causing maturation defects in NK cells (219). The MYSM1 histone H2A deubiquitinase also contributed to the development of ILC1s in other organs. In addition to modifying histones, MYSM1 also functions as a transcriptional regulator of ID2 expression during the maturation of NK cells by recruiting NFIL3 to the *Id2* gene locus. MYSM1 was involved in maintaining an active chromatin configuration at the *Id2* locus (219), further promoting its expression. Another epigenetic mechanism that regulates NK cell development involves repressive histone marks such as the tri-methylation of lysine residue 27 of Histone 3 protein during early NK cell differentiation (220). In the absence of this marker through the repression of EZH2 enzymatic activity (enhancer of zeste homolog 2), ILC1 and NK cell lineage commitment was enhanced, together with increased NK cell survival and NKG2D-mediated cytotoxicity (220).

Apart from the regulation of gene expression on the transcriptional level, another epigenetic mechanism is required for the proper development of ILC1s and the adequate maturation of NK cells. Available data implicate small non-coding RNA molecules (221–226), such as microRNAs (miRs), to regulate posttranscriptional gene expression by binding to the 3′ untranslated region (UTR) of mRNAs and inducing either suppression or mRNA translation or its degradation (227). Deletion of the RNase III enzyme Dicer-1, an enzyme required for the generation of single-stranded 20–25 bp long non-coding RNA molecules, in NKp46-expressing cells revealed the role of miRs in murine NK cells and ILC1s (223). The number of NK1.1^+^ cells in the organs of Dicer-1 mutant mice was affected, along with the impaired maturation of NK cells. NK cells without miR showed a diminished function, including reduced target cell cytotoxicity and IFN-γ production. Additionally, in Dicer-1-deficient mice, the IL-15 receptor signaling in NK cells was impaired. This finding explains, at least in part, the decreased survival of NK cells and the observed perturbations in NK cell maturation.

The effects of single miRs, such as miR142, miR155, miR150, and miR15/16, revealed specific effects and potential target genes. The conserved *miR142* sequence encodes two highly expressed mature miRNAs, 142-3p and 142-5p, which have different mRNA targets (221). The target of the miR142-3p is the 3′ UTR of *Itgav* gene that encodes integrin-α_V_. In the absence of miR142-3p, this integrin was up-regulated in ILC1s and promoted their survival. The other product of the *miR142* sequence, miR142-5p, was targeting the 3′ UTR *suppressor of cytokine signaling 1* (*Socs1*) gene, a negative regulator of IL-15 signaling. Thus, in the absence of miR142-5p, SOCS1 un-antagonized, leading to impaired IL-15 signaling (221).

In humans, miR155 was shown to down-regulate SH2 containing 5′ inositol phosphatase (SHIP1), which in part contributes to the regulation of IFN-γ production following stimulation (225). In mice, miR155 targeted the 3′ UTR of *Noxa* transcripts during homeostasis and of *Socs1* transcripts during the activation of NK cells (226). The direct functional target of miR-150 and miR15/16 was the TF c-Myb, through which the maturation program was controlled (222, 224).

## Plasticity Toward Group 1 ILCs

Although ILCs comprise separate lineages of innate lymphocytes defined by distinct lineage-specifying TFs, a considerable amount of plasticity after fate commitment was reported for most ILC lineages in mice and humans, often connected to a certain tissue microenvironment or in the context of inflammation (228). Plasticity is characterized by the down-regulation of lineage-specifying TFs, such as RORγt for ILC3s or GATA-3 for ILC2s, and acquisition of master TFs of alternative cell fates, acquisition of phenotypic characteristics of other ILC lineages (e.g. up-regulation of NK cell receptors), and production of cytokines not associated with the original lineage. The plasticity of ILCs was first described for ILC3s (52, 229). Fate-labeling for RORγt expression revealed that ILC3s were able to differentiate into cells phenotypically resembling ILC1s (referred to as ex-RORγt^+^ ILC3s or ex-ILC3s) (52, 229–231). This process was accompanied by the gradual up-regulation of ILC1 signature genes such as T-bet, NK receptors (NKp46, NK1.1, and NKG2D), and cytokine receptors (IL12Rβ2), as well as effector functions (19, 21, 44, 53, 55). During this process, ex-ILC3s became IFN-γ-producing lymphocytes, which were responsive to several cytokines, including IL-12 and IL-23, and promoted inflammation and immunopathology in experimental models of colitis and *Salmonella enterica* infection (44, 52, 207, 229, 232, 233). T-bet deficiency was *vice versa* reported to promote colitis in response to *Helicobacter typhlonius* that was mediated by IL-17A-producing ILC3s (234, 235). In humans, differentiation of ILC3s toward CD127^+^ ILC1s was described in the intestine of patients with Crohn's disease and was promoted by cytokines IL-2 and IL-12 and CD14^+^ DCs. Interestingly, this process was found to be reversible and stimulated by IL-1β, IL-23, retinoic acid, and CD14^−^ DCs (19, 229, 230). Data obtained in fate-labeling studies in mice using either *ROR*γ*t*^Cre^ (230) or *NKp46*^Cre^ (236) also support the model that the plasticity of ILC3s is a reversible process. Signals regulating NKp46 expression on CCR6^−^ ILC3s included the Notch-T-bet axis as a positive regulator and TGF-β signaling as a negative regulator (236). Altogether these studies provide evidence for the reversible plasticity of CCR6^−^ ILC3s toward ILC1s, mediated by signals that regulate RORγt and T-bet.

While the down-regulation of RORγt and the up-regulation of T-bet occurs at steady state in CCR6^−^ ILC3s, the plasticity of NK cells or ILC2s might require a trigger, such as chronic inflammation. The conversion of ILC2s to an ILC1-like phenotype is triggered by cytokines, such as IL-1, IL-12, and IL-18, and was described in the context of chronic obstructive pulmonary disease (237–240). This process is connected to the up-regulation of T-bet, and the genetic deletion of T-bet using *NKp46*^Cre^ resulted in enhanced ILC2 responses, suggesting that the balance of the lineage-specifying TFs GATA-3 and T-bet determines ILC2 plasticity (241).

Whether the conversion of NK cells to ILC1-like cells is occurring at a steady state is difficult to evaluate because of the lack of fate-labeling studies for the NK lineage-specifying TF EOMES. However, fate-labeling was carried out using Cre under the NKp46 promoter, which is expressed in NK cells, ILC1s, CCR6^−^ ILC3s, and subsets of γδ T cells (138). While the down-regulation of NKp46 was described for ILC3s (236), the results did not provide evidence that NK cells down-regulate NKp46 at steady state (138). It should be considered though that conversion in other ILC lineages, which also express NKp46, would not be detected using this fate-labeling strategy. The first evidence for the potential conversion of NK cells into ILC1-like cells came from studies that investigated the unusual subsets of ILC1s in the salivary gland. Unlike ILC1s in other organs, the salivary gland ILC1s co-expressed EOMES and T-bet but did not developmentally depend on either of these TFs and also not on NFIL3, suggesting that they have different developmental requirements (199, 242). In addition, the salivary gland ILC1s depicted hallmarks of tissue-resident cells, such as TGF-β imprinting, that was also reported for ILC1s in different organs, for instance, the intestine (20). ILC1s in the salivary gland were reduced in the absence of TGF-β signaling, and the phenotypical markers of ILC1s, such as CD49a and TRAIL were down-regulated, whereas EOMES was up-regulated (199). Furthermore, NK cells that were hyper-responsive to TGF-β, due to the genetic manipulation of TGF-βRI or deletion of SMAD4, developed an ILC1-like phenotype in the salivary gland or within tumor tissue. As a consequence, these ILC1-like NK cells failed to control tumor growth or viral infection with cytomegalovirus (181, 182). Some of the effects that TGF-β has on the NK/ILC1 fate decisions are mediated *via* the balance of the master TFs T-bet and EOMES. Notably, it was reported that the forced expression of EOMES driven by the T-bet promoter turned ILC1s into cells with NK cell properties (178). However, it remains unclear whether this occurs *in vivo*, and if yes, under which conditions.

While ILC plasticity after lineage commitment is now well-established to occur, additional investigation is required to elucidate how the plastic behavior of ILCs could be therapeutically harnessed.

## Specific Targeting to Uncover Functional Specialization of Group 1 ILC Subsets

Despite progress in the generation of genetically modified mice, specific targeting of ILCs remains a major challenge in the field because of the large overlap in gene expression between ILCs and T cells as well as other immune cells. Since a systematic review of genetic models for the investigation of ILC function was recently published (243), we aim to focus the discussion on NK cell receptor (NKR)^+^ ILCs that comprise conventional NK cells, ILC1s, and CCR6^−^ ILC3s. Concerning NKR^+^ ILCs, specific targeting of each subset is further complicated by shared receptors such as NKG2D, NKp46, and NK1.1 and TFs such as T-bet, NFIL3, or TOX within NKR^+^ ILCs, making them alone not a suitable target (141). While antibody-mediated depletion strategies using αNK1.1 or αThy1 were effective, more specific depletion strategies were developed using genetic models based on *NKp46*^Cre^ mice (138, 153, 244). While NKp46 is fairly specific to group 1 ILCs, a second allele is required to ensure specificity among group 1 ILCs, which is often a floxed mouse for an essential TF such as EOMES or RORγt. Following the targeting strategy, the generation of *NKp46*^Cre^ *Eomes*^fl/fl^ resulted in the selective ablation of NK cells, thus allowing a definitive conclusion about the contribution of NK cells in an experimental autoimmune encephalomyelitis model (178, 179). *NKp46*^Cre^ *Rorc*(γt)^fl/fl^ mice were likewise generated to investigate redundant and non-redundant functions of ILC3s during *Citrobacter rodentium* infection and in colitis models (233, 245). While these two strains provide specific targeting for NK cells and CCR6^−^ ILC3s, respectively, the genetic mouse models for ILC1s are even more difficult to develop. *NKp46*^Cre^ *Tbx21*^fl/fl^ mice lacked ILC1s (179), but a contribution of NK cells or CCR6^−^ ILC3s in these mice could not be excluded because NK cells and CCR6^−^ ILC3s have a migration or maturation defect in T-bet-deficient mice. It should be also considered that the phenotype might be dependent on which line of NKp46^Cre^ deleter mice is used (179, 241). Furthermore, mice deficient for the TF HOBIT were used to investigate ILC1 function in the liver because ILC1s, but not NK cells, are reduced in the liver of these mice. However, the use of this mouse line is limited to TRAIL^+^ NK cells and not ILC1s in other organs (210, 213). Therefore, the goal for *NKp46*^Cre^ to delete a selective TF important for ILC1 subsets in many organs is still not achieved.

## Regulation of NK Cell Development and Function by Receptor–Ligand Interaction

The activation of NK cells is mediated to a large degree by the integration of stimulatory and inhibitory signals as measured by the engagement of NK receptors by its cognate ligands. NK cells need to be calibrated during development to become activated if a defined threshold of stimulatory to inhibitory signals is exceeded, a process coined “NK cell education” or “licensing.” Classical NK cell education is linked to self recognition and mediated by inhibitory receptors for class I MHC, such as Ly49 receptors or KIR (82, 246–248). Thus, this process requires the timely expression of the corresponding ligands for the receptors involved in the education process. Moreover, besides the type of MHC molecule expressed and the type of receptors on NK cells, the strength of class I MHC–Ly49 receptor interaction also defines the quality and the quantity of NK cell education (249). In connection with this, it was observed that the absence of MHC I on the surface of cells, by the genetic deletion of β_2_-microglobulin, TAP, or K^b^D^b^, resulted in the hypo-responsiveness of NK cells (250–252). In line with these findings, NK cells with mutations in ITIMs required for inhibitory signaling were functionally impaired. Further, the deletion of intracellular downstream signaling molecules, SH-2-domain-containing protein tyrosine phosphatase 1 (SHP1) and SH-2-domain-containing inositol-5-phosphatase (SHIP), resulted in the hypo-responsiveness of NK cells (253–255). On a molecular level, the MHC I education process was linked to the reorganization of the nanostructure of immunoreceptors and confinement in domains, thus generating the basis for different activation thresholds (251, 252, 256).

Interestingly, Ly49s were strongly underrepresented but not totally absent on ILC1s, suggesting differences between NK cell and ILC1 education. However, with respect to ILC1s, fundamental questions remain unanswered. These questions include whether ILC1s require an education process at all and, if so, whether the education is regulated by cell-bound immunoreceptor–ligand interaction. If this is true, how much of education is regulated by inhibitory receptors such as CD94/NKG2A expressed by ILC1s? Due to the lack of data for ILC1 education, we focus on the regulation of NK cell development and activation (21, 141).

NK cell education not only is limited to self recognition of MHC I molecules but also involves self recognition of non-MHC ligands such as those provided by the receptor–ligand pairs CD155-TIGIT, CD48-2B4, and CLR-b-NKRP1-B (252). Moreover, it became apparent that stimulatory receptors mediating induced-self recognition are involved in the NK cell education process as well. NKG2D is a stimulatory receptor, which recognizes induced-self ligands and which regulates NK cell education (90). NKG2D is already expressed early on from the NKP stage. For induced-self ligands, it is however incompletely understood when these ligands are expressed under homeostatic conditions and which cell types would be involved in this process (132, 257, 258). Parallel to the finding that NK cells from MHC I-deficient mice were hypo-responsive, a similar paradoxical phenotype was uncovered for NKG2D-deficient mice having hyper-responsive NK cells despite the lack of an important stimulatory NK cell receptor. Notably, mice deficient for NKG2D (*Klrk1*^−/−^) were hyper-responsive, resulting in the superior control of MCMV infection and tumor growth (257, 258). However, immunosurveillance of tumors expressing NKG2D ligands was impaired in NKG2D-deficient mice (259). Mechanistically, NKG2D regulated signaling *via* the natural cytotoxicity receptor NKp46 and the signaling molecule CD3ζ (258). While the precise timing of NK cell education is not well-defined, some studies suggested that NK cell education is not limited to a time window during development but represents a continuous process. This is supported by studies that used adoptive transfer of uneducated NK cells in MHC I-sufficient hosts that could restore NK cell functionality (260, 261). In addition, data obtained in models that overexpressed ligands for stimulatory receptors such as m157 or NKG2D ligands, in which the NK cells were persistently exposed to a receptor engagement, revealed that NK cells adapted to this stimuli, for instance by the down-regulation of the stimulatory receptor (262–264). Although it remains elusive if the adaptation of NK cell reactivity to sustained activation by stimulatory ligands due to overexpression is the underlying similar mechanism described for NK cell education, the findings become relevant in the context of anti-tumor immunity where, for instance, NKG2D ligands might be continuously expressed or shedded from the tumor cells, thus saturating their receptors. Although it is controversial if the chronic expression or shedding of NKG2D ligands should be regarded as a tumor escape mechanism or if it is promoting tumor immunosurveillance, these findings indicate the importance of the regulation of NK cell activity by receptor–ligand interaction (265–267). Apart from the chronic expression of induced-self ligands on tumor cells, blocking antibodies for inhibitory receptors targeting KIR or NKG2A are evaluated in clinical trials to promote anti-tumor immunity (267). The blockade of inhibitory receptors on NK cells has the potential to complement T cell immunotherapy because the efficiency of T cell checkpoint blockade correlated with the production of neoantigens by tumor cells present on MHC I. However, the tumor cells that did not produce neoantigens or escaped MHC I-peptide recognition by CD8^+^ T cells (268) could be recognized and lysed by NK cells expressing inhibitory receptors to detect the presence of MHC I. However, data available so far might indicate that there is a narrow therapeutic window defined by the blocking of the inhibitory receptor and the effects on NK cell education, rendering the cells hypo-responsive (267).

In summary, NK cells need education mediated by the engagement of inhibitory and stimulatory receptors during development. NK cell education is required for both adequate reactivity and tolerance toward self. Blocking of inhibitory NK cell receptors during anti-tumor therapy can complement checkpoint blockade and illustrates the transfer of basic knowledge for human therapy.

## Author Contributions

All authors listed have made a substantial, direct and intellectual contribution to the work, and approved it for publication.

## Conflict of Interest

The authors declare that the research was conducted in the absence of any commercial or financial relationships that could be construed as a potential conflict of interest.
